# Development of a microencapsulated probiotic containing *Pediococcus acidilactici* WU222001 against avian pathogenic *Escherichia coli*

**DOI:** 10.14202/vetworld.2023.1131-1140

**Published:** 2023-05-30

**Authors:** Watcharapong Mitsuwan, Phirabhat Saengsawang, Juthatip Jeenkeawpieam, Veeranoot Nissapatorn, Maria de Lourdes Pereira, Warangkana Kitpipit, Thotsapol Thomrongsuwannakij, Saranporn Poothong, Sasi Vimon

**Affiliations:** 1 Akkhraratchakumari Veterinary College, Walailak University, Nakhon Si Thammarat, 80160, Thailand; 2 One Health Research Center, Walailak University, Nakhon Si Thammarat, 80160, Thailand; 3 Center of Excellence in Innovation of Essential Oil and Bioactive Compounds, Walailak University, Nakhon Si Thammarat, 80160, Thailand; 4 School of Allied Health Sciences, Southeast Asia Water Team, World Union for Herbal Drug Discovery, and Research Excellence Center for Innovation and Health Products, Walailak University, Nakhon Si Thammarat, Thailand; 5 CICECO-Aveiro Institute of Materials and Department of Medical Sciences, University of Aveiro, 3010-193 Aveiro, Portugal; 6 Food Technology and Innovation Center of Excellence, Walailak University, Nakhon Si Thammarat, 80160, Thailand; 7 Department of Animal Husbandry, Faculty of Veterinary Science, Chulalongkorn University, Bangkok, 10330, Thailand

**Keywords:** antibacterial activity, avian pathogenic *Escherichia coli*, microcapsule, microencapsulation, *Pediococcus acidilactici*, probiotics

## Abstract

**Background and Aim:**

Probiotics are beneficial microorganisms for humans and animals. In this study, we developed a microencapsulated probiotic with antibacterial activity against avian pathogenic *Escherichia coli* (APEC).

**Materials and Methods:**

Alignment of the 16S rRNA sequences of the isolate WU222001 with those deposited in GenBank revealed that the isolate was *Pediococcus acidilactici* with 99.6% homology. This bacterium was characterized as a probiotic based on its tolerance toward *in vitro* gastrointestinal tract (GIT) conditions, hydrophobicity, and auto-aggregation. The antibacterial activity of the probiotic’s culture supernatant against APEC was investigated using a broth microdilution assay. *Pediococcus acidilactici* was microencapsulated using sodium alginate and agar with diameters ranging from 47 to 61 μm. Then, physicochemical characteristics and stability of the microcapsules were determined.

**Results:**

The isolate was characterized as a probiotic based on its resistance to low pH, bile salts, and pancreatin, with relative values of 79.2%, 70.95%, and 90.64%, respectively. Furthermore, the bacterium exhibited 79.56% auto-aggregation and 55.25% hydrophobicity at 24 h. The probiotic’s culture supernatant exhibited strong antibacterial activity against clinical APEC isolates with minimum inhibitory concentration and minimum bactericidal concentration of 12.5% and 25% v/v, respectively. Microencapsulation-enhanced bacterial viability in GIT compared to free cells. Moreover, 89.65% of the encapsulated cells were released into the simulated intestinal fluid within 4 h. The viable count in microcapsules was 63.19% after 3 months of storage at 4°C.

**Conclusion:**

The results indicated that the culture supernatant of *P. acidilactici* inhibited the growth of APEC. In addition, microencapsulation extends the viability of *P. acidilactici* under harsh conditions, indicating its potential application in the feed production.

## Introduction

*Escherichia coli* is a commensal Gram-negative bacterium found in the intestinal tract of humans and animals. However, some *E. coli* strains are pathogenic and can cause fatal illnesses in humans and animals [1]. Avian pathogenic *E. coli* (APEC) causes colibacillosis [2] and several other symptoms in poultry, such as perihepatitis, airsacculitis, pericarditis, egg peritonitis, salpingitis, coligranuloma, omphalitis, cellulitis, and osteomyelitis [3]. As APEC infections decrease meat and egg production [4], APEC is considered an important pathogen linked to economic losses in the global poultry production. Importantly, it is also a potential foodborne zoonotic pathogen that is an external source of human extraintestinal infections [5]. Furthermore, treatment of colibacillosis is challenging because of increasing antibiotic resistance in APEC [6]. Hence, a novel therapeutic is urgently required to treat colibacillosis caused by APEC.

Probiotics, such as lactic acid bacteria (LAB), are beneficial microorganisms found in several parts of the human and animal bodies, including the digestive tract, oral cavity, and reproductive systems [7]. *Pediococcus acidilactici* is used in fermentation as a probiotic and biopromoter of animal growth. Lactic acid bacteria are being increasingly used in traditional fermented and modern processed foods [8]. The genus *Pediococcus* spp., including *P. acidilactici*, produce antimicrobial peptides called bacteriocins used in food and health industries [8]. Recently, consuming yogurt containing *P. acidilactici* has been shown to lower copper and nickel levels in the blood more effectively than conventional yogurt [9]. Previous study has isolated *P. acidilactici* from fermented foods as it is generally used as a starter [8]. This study focused on BX1, a supplemented animal feed containing several microorganisms, such as *Pediococcus* spp., *Pichia* spp., and *Dekkera* spp. These microorganisms are characterized as probiotics, targeting the gut of humans and other animals. However, the survival rate of free probiotic cells is low due to gastrointestinal tract (GIT) conditions, such as low pH, enzymes, and bile salts. Therefore, a novel strategy is required to enhance the survival rate of probiotics for industrial applications.

Microencapsulation is the process of creating microcapsules with active microorganisms covered with polymers and organic and inorganic compounds. Therefore, it is a practical technique to facilitate probiotic activity [10]. Probiotics can be effectively delivered into the body using microcapsules with minimal damage due to GIT conditions [10]. Furthermore, microcapsules can control the release of probiotics, ensuring their successful delivery to the site of action [11]. Furthermore, encapsulation maintains the viability of probiotics during food manufacturing and long-term storage [11].

To the best of our knowledge, this study is the first to report a probiotic against APEC. This study aimed to isolate and characterize the probiotic bacteria BX1, which produces antimicrobial compounds against APEC. We evaluated the probiotic properties, including tolerance to the GIT conditions, hydrophobicity, auto-aggregation, and antibacterial activity of the isolated bacteria. Moreover, we prepared and characterized microencapsulated *P. acidilactici*.

## Materials and Methods

### Ethical approval

The study did not involve any live animals or humans, so ethical approval was not necessary.

### Study period and location

The study was conducted from April 2022 to December 2022. Isolation, identification, antibacterial activity tests, and microencapsulation were investigated at Walailak University, Nakorn Si Thammarat, Thailand.

### Isolation, identification, and culture of bacteria

Ten grams of BX1 powder (BIOFEED (Thailand) Co., Ltd.) were enriched in 90 mL of Mann Rogosa sharpe (MRS) broth (HiMedia, India), incubated at 37°C for 24 h, and streaked onto MRS agar. A single colony was inoculated into the MRS broth (HiMedia) containing 20% glycerol at −80°C until further use. We cultured three clinical APEC isolates, *E. coli* ATCC 25922, *Bacillus cereus* WU22001, and *Staphylococcus aureu*s ATCC25923, in tryptic soy broth (HiMedia). We identified the LAB by evaluating gram staining and catalase activity. Subsequently, the genomic DNA of the WU222001 strain was extracted and purified as described by Mitsuwan *et al*. [12]. The 16S rRNA gene was amplified by PCR and sequenced (Macrogen, Republic of Korea) using the universal primers: 27F (5′-AGAGTTTGATCMTGGCTCAG-3′), 1492R (5′-TACGGYTACCTTGTTA CGACTT-3′), 518F (5′-CCAGCAGCCGCGGTAATACG-3′), and 800R (5′-TACCAGGGTATCTAATCC-3′). Then, the 16S rRNA gene sequences were aligned and analyzed with the EzBioCloud database (http://www.ezbiocloud.net/eztaxon) [13]. The sequencing-matching program for genus and species was analyzed using the Clustal_X program [14]. The phylogenetic tree was reconstructed based on the neighbor-joining method in Mega 7.0 (https://www.megasoftware.net/) [15] by bootstrap resampling with 1000 replicates [16].

### Tolerance to GIT conditions

The tolerance of the isolate to acidic pH, bile salts, pepsin, and pancreatin was studied as described by Somashekaraiah *et al*. [17]. We first centrifuged 1 mL of the overnight culture at 8,000× *g* for 5 min, resuspended in phosphate-buffered saline (PBS; pH = 7.2), and adjusted to 0.5 McFarland standards. Then, 1 mL of each sample was centrifuged at 8000× *g* for 5 min to collect cell pellets. The strains were inoculated in MRS broth and adjusted to pH 3 by 1 N hydrochloric acid. We also evaluated the tolerance of the isolate toward pepsin (3 g/L, pH = 2.5), pancreatin (1 g/L, pH = 8), and bile salt (3 g/L). Samples were incubated at 37°C for 3–4 h. The survival rate was investigated on MRS agar after incubation at 37°C for 24 h and calculated as described by Somashekaraiah *et al*. [17]:

Survival rate (%) = [Final (Log colony-forming unit [CFU]/mL)/Initial (Log CFU/mL)] × 100.

### Hydrophobicity and auto-aggregation

Hydrophobicity and auto-aggregation were determined as described by Somashekaraiah *et al*. [17]. Bacterial suspensions grown overnight in MRS broth were harvested by centrifugation at 8000× *g* for 10 min. To determine the hydrophobicity, the bacterial cells were washed twice using PBS and resuspended in PBS containing 0.1 mL of hexadecane (A0). Hydrophobicity was determined by measuring the optical density (OD_600_) of the aqueous phase at 600 nm (A1). For auto-aggregation, bacterial cells were adjusted to an OD_600_ value of 0.8–1. The suspension was then incubated at 37°C for 0, 2, 5, and 24 h. Autoaggregation at different time points was determined by measuring the OD_600_ of the aqueous phase (A time). The values of hydrophobicity and auto-aggregation were calculated according to the following formulas as described by Somashekaraiah *et al*. [17]:

Hydrophobicity (%) = [(1 − A_1_)/A_0_] × 100

Autoaggregation (%) = [1 − (A_time_)/A_0_] × 100

### Antibacterial activity of the probiotic supernatant against APEC

The antibacterial activity of the probiotic supernatant against APEC was investigated using agar well diffusion and broth microdilution assays as described by Mitsuwan *et al*. [12]. *Pediococcus pentosaceus* was cultured in an Mueller–Hinton broth (MHB) medium and incubated at 37°C for 24 h. The sample was centrifuged at 5,000× *g* for 5 min, filtered through a 0.45 μm sterile filter (Pall Corporation, USA), and stored at −20°C until use. All pathogens were cultured in MHB, incubated for 4–6 h at 37°C, and then adjusted to 0.5 McFarland standards. After spreading the samples on Mueller–Hinton agar plates, 6-mm-diameter wells were cut on the agar surface using the back of a sterile tip. Each well was filled with 100 μL of the probiotic culture supernatant. The culture medium and 3% hydrogen peroxide were used as positive and negative controls, respectively. The samples were incubated at 37°C for 24 h, and zones of inhibition were measured using a Vernier caliper.

The minimum inhibitory concentration (MIC) and minimal bactericidal concentration (MBC) of the supernatant against the pathogens were determined by broth microdilution assay. Briefly, 100 μL of the filtered supernatant was added to a 96-well microtiter plate containing 100 μL of MHB and serially diluted two-fold using MHB. The pathogens (100 μL, 10^6^ CFU/mL) were added to each well and incubated at 37°C for 18 h. Cefotaxime and vancomycin were used as positive controls, while 1% dimethyl sulfoxide was the negative control. After that, 0.03% resazurin (Thermo Fisher Scientific, UK) was added and incubated for 3 h to determine the MIC, which was defined as the lowest concentration that inhibited bacterial growth, indicated by a blue coloration. The MBC values of all blue-colored wells were then checked by streaking the culture from the wells onto Trypticase Soy agar plates.

### Encapsulation of the probiotic bacteria

#### Preparation of P. acidilactici WU222001 (PAWU222001) encapsulated agar-alginate (AG-AL) microcapsules (PAWU/AG-AL)

**Figure-1:**
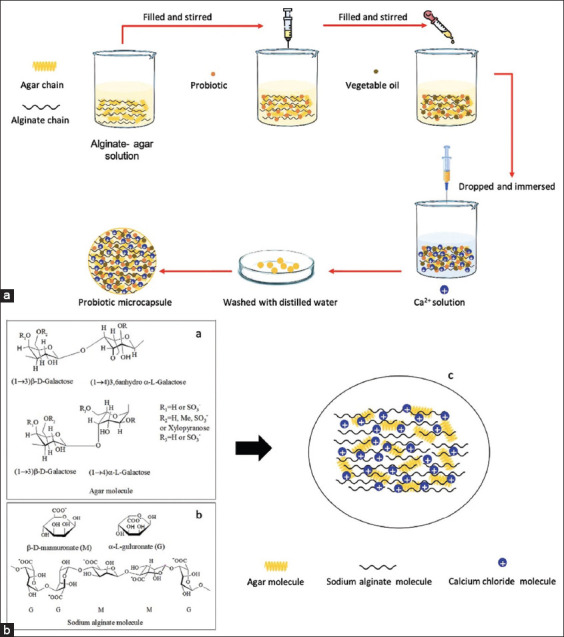
(a) Schematic for steps of preparation of PAWU/agar-alginate (AG-AL), (b) molecular structure of AG and AL and their ideal gelation mechanism diagram.

We dissolved 2 g sodium AL (Sigma-Aldrich, USA) and 0.2 g AG (Patanasin enterprise, Thailand) in 100 mL of deionized water (DI). After adding Tween 80 (2 mL), the mixture was stirred for 1 h at 90°C. The AG-AL solution was maintained under constant stirring for 20 min. Then, PAWU222001 (20 mL) and soybean oil (20 mL) were added dropwise through a needle (diameter 1.8 mm) from a distance of 5 cm into aqueous CaCl_2_ solution (0.1 mol/L) (Merck, Germany) to obtain the microcapsules. Finally, the PAWU222001 encapsulated AG-AL solution, namely, PAWU/AG-AL, was sieved by a mesh (diameter 0.053 mm) and washed thrice with DI water before drying in a hot air oven at 30°C overnight (Figure-1a).

### Determination of physicochemical characteristics

#### Microcapsule morphology

The shape and surface morphology of AG-AL and PAWU/AG-AL were determined using a scanning electron microscopy (SEM)-IT300 SEM (Zeiss, Germany) using the cross-sections of PAWU/AG-AL obtained using a razor blade.

#### Structural characterization of microcapsules

The chemical structures of the microcapsules, that is, AG-AL and PAWU/AG-AL, were identified using a Bruker Alpha Fourier-transform infrared spectrometer (FTIR) using the attenuated total reflection technique in the range of 4000 cm^−1^–400 cm^−1^.

#### Viability of microcapsule under pelleting temperature

The thermal stability and components of the microcapsules were evaluated after heating at 75°C, 80°C, 85°C, 90°C, 95°C, and 100°C for 3 min.

#### Swelling and cumulative release studies

The swelling of PAWU/AG-AL was determined in the simulated digestive tract according to the protocol of Mokarram *et al*. [18]. Simulated gastric fluid (SGF) was prepared by dissolving 2 g of sodium chloride in DI water, adding 7 mL of concentrated hydrochloric acid, and adjusting to pH = 1.2 with 0.1 M hydrochloric acid to a final volume of 1 L. Simulated intestinal fluid (SIF) was prepared by dissolving 6.8 g of dipotassium phosphate in 190 mL of 0.1 M sodium hydroxide. The pH was adjusted to 7.5 before adjusting the final volume to 1 L. For the simulated gastric stage study, PAWU/AG-AL (1 g ± 0.05 g) was added to the SGF (100 mL). The pH of the solution was adjusted to 2.5, and the PAWU/AG-AL was incubated at 39.5°C ± 0.5°C in a thermostat water bath for 20–180 min with 20 min intervals. After incubation at each point, PAWU/AG-AL was filtered and weighed while the SGF solution was collected for use in the intestinal stage. For the simulated intestinal stage study, solutions containing trypsin (2 mg/mL, 1 mL), bile (40 mg/mL, 14 mL), pancreatin (3.2 mg/mL, 7.5 mL), and SIF (7.5 mL) were mixed with the above SGF solution. The pH of SIF was adjusted to 8, while the PAWU/AG-AL was incubated at 39.5°C ± 0.5°C for 200, 220, and 240 min. The treated PAWU/AG-AL was collected and weighed at each incubation time. The percentages of swelling were calculated according to the following formula as described by Mokarram *et al*. [18].

DS = [(Ws − W_0_)/W_0_] × 100

Where W_0_ and W_s_ are the weights of the dry and the swollen microcapsules after 4 h, respectively.

The release behavior of PAWU/AG-AL was investigated as described by Gbassi *et al*. [19]. After incubation at each point, 1 mL of supernatant was withdrawn, and the released bacteria were determined using the pour plate technique in MRS agar. The index of cell release was calculated using the following equation.


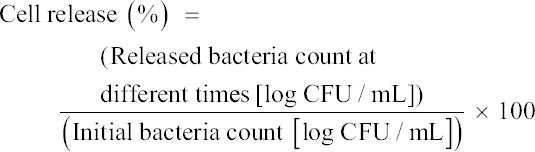


### Determination of microcapsule stability

#### Encapsulation efficiency (EY)

Encapsulated PAWU222001 in AG-AL microcapsules were analyzed by homogenizing the filtered microcapsules (1 g) in 10 mL of sodium citrate. PAWU222001 strain was counted on MRS agar, and the EY% was calculated as described by Gbassi *et al*. [19].

EY (%) = (N/N_0_) × 100

Where, N is the number of viable entrapped cells released from the microcapsules, and N_0_ is the number of free cells added to the PAWU/AG-AL microcapsules.

#### Effect of acids, enzymes, and temperature on the viability of free cells and PAWU/AG-AL

We tested the tolerance characteristics of PAWU/AG-AL according to Mokarram *et al*. [18] with minor modifications. We prepared the acid, bile salt, pepsin, and pancreatin as described previously. The samples were investigated by soaking PAWU/AG-AL (5 ± 0.5 g) and free cells into the solutions.

Then, all tubes were incubated in a thermostatic water bath vibrator at 39.5°C ± 0.5°C for 30 min. For thermal treatment, PAWU/AG-AL and free cells were tested at 85°C for 1 min, immediately removed, and their viability was measured as described by Gbassi *et al*. [19].

#### Impact of storage condition

The storage stability of PAWU/AG-AL was studied according to Mokarram *et al*. [18]. Briefly, free PAWU222001 (10 mL) and PAWU/AG-AL (10g ± 0.5 g) were separately sealed in glass vials and wrapped with aluminum foil. The containers were stored at room temperature (28°C) for 90 days. Both samples were collected after 30, 60, and 90 days of storage to determine the viability.

### Statistical analysis

Storage stability and tolerance were analyzed using a one-way analysis of variance (the Statistical Package for the Social Sciences Inc., Chicago, IL, USA) in a completely randomized design. The mean was evaluated using Duncan’s new multiple-range *post hoc* test. The statistical significance was considered at p < 0.05.

## Results and Discussion

### Identification of probiotic bacteria

**Figure-2:**
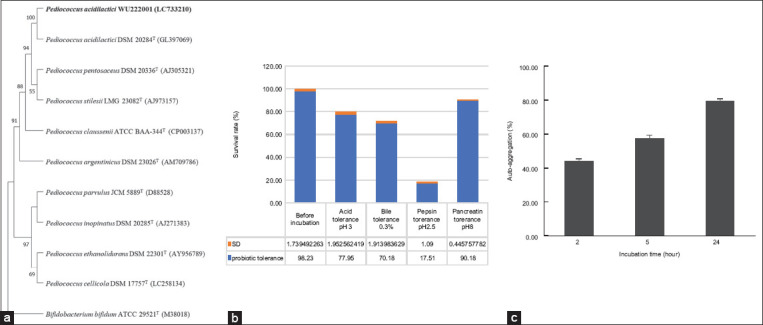
A phylogenetic tree of PAWU222001 and their related type strains based on 16S rRNA gene sequences. The neighbor-joining method was used to construct phylogenetic tree. The percentage of replicate trees in which the associated taxa clustered together in the bootstrap test (1000 replicates) is shown next to the branches. Bar represents 0.002 substitutions per nucleotide position. Strain WU222001 is highlighted in bold. (a) *Bifidobacterium bifidum* ATCC 29521^T^ was represented for outgroup, (b) survival rate (%) of the selected PAWU222001 under gastrointestinal tract conditions, (c) auto-aggregation (%) of PAWU222001 isolates at 2, 5, and 24 h. The data are presented as mean ± standard deviation.

The isolate was identified as a Gram-positive, non-endospore-forming, and catalase-negative bacteria. Based on 16S rRNA sequence analysis, the isolate was identified as *P. acidilactici* (1497 bp; Accession No. LC733210) with 99.6% homology with *P. acidilactici* DSM 20284^T^ (Accession No. GL397069) from GenBank (Figure-2a). These findings showed that the homology of these bacteria was not 100%, indicating that they may be different strains [12].

### Characterization of probiotics

*Pediococcus acidilactici* is found in the gut microbiota of humans and animals. It can be applied during fermentation as an animal growth biopromoter serving as a probiotic [8]. To characterize *P. acidilactici* WU222001 as the probiotic, we determined this bacteria’s tolerance toward conditions similar to the digestive tract, hydrophobicity, and auto-aggregation. The isolates demonstrated relatively strong resistance to pancreatin with a percentage survival rate of 90.64% (Figure-2b). In addition, 79.2% and 70.95% were resistant to pH = 3 and bile salts, respectively. We also observed that the cells exhibited 55.25% hydrophobicity. The auto-aggregation ability of *P. acidilactici* significantly increased with an increase in the incubation period (p < 0.05) (Figure-2c). Furthermore, the bacteria exhibited 79.56% auto-aggregation after 24 h. Similarly, *P. acidilactici* NCDC252, a probiotic candidate, could survive in intestinal conditions and exhibited aggregation and adhesion capabilities [15]. The auto-aggregation of probiotics indicates their ability to adhere and colonize in the gut [20]. Furthermore, *P. acidilactici* adheres to the intestinal epithelial cells of the human GIT, stabilizing tract stability, and preventing intestinal infections [21]. Unfortunately, the bacteria exhibited low cell viability in the presence of pepsin. Therefore, a modified microencapsulation strategy must be established to protect against bacterial damage by GIT conditions.

### Antimicrobial activity of PAWU222001

The antibacterial activity of the probiotic supernatant against pathogens, including APEC, *E*. *coli*, *B*. *cereus*, and *S*. *aureus*, was represented by the zone of inhibition, MIC, and MBC values. The results showed that the inhibition zone of *P. acidilactici* supernatant against APEC ranged between 12 ± 0.00 and 15.33 ± 1.15 mm (Table-1). Furthermore, the supernatant inhibited the growth of *E*. *coli*, *B*. *cereus*, and *S*. *aureus* (Table-1). The MIC and MBC values of the *P. acidilactici* supernatant against the clinical APEC isolates were 12.5% and 25% v/v, respectively, which was similar to the MIC and MBC values against the other tested pathogens. Therefore, the MBC/MIC ratio demonstrated that the supernatant has bactericidal activity against APEC [1]. Probiotics, including *P. acidilactici*, might be viable alternatives to chemical drugs as they can operate antagonistically against foodborne infections. *Pediococcus acidilactici* has been reported to produce bacteriocins, which are antimicrobial peptides used in the food and pharmaceutical industries [8]. Bacteriocin induces pore formation in the bacterial cell membrane, resulting in bacterial cell lysis [22]. *Pediococcus acidilactici* also produces powerful antifungal metabolites that can inhibit mycotoxin-inducing molds [22].

**Table-1:** Antibacterial activity of the culture supernatant of *Pediococcus acidilactici* WU222001 against clinical isolates Of Apec.

Bacterial strains	Inhibition zone (mm) (Mean ± SD)		MIC/MBC (%v/v)	
	
	Supernatant	Ampicillin	Supernatant	Antibiotics (µg/mL)
APEC CH01	15.33 ± 1.15	R	12.5/25	0.125/0.250^a^
APEC CH06	12.00 ± 0.00	R	12.5/25	0.250/0.500^a^
APEC CH08	12.50 ± 0.50	R	12.5/25	0.500/0.500^a^
APEC CH09	15.00 ± 0.00	R	12.5/25	0.250/0.250^a^
APEC CH10	13.33 ± 0.29	R	12.5/25	0.250/0.250^a^
*Escherichia coli* ATCC 25922	15.00 ± 0.00	S	12.5/25	0.250/0.250^a^
*Bacillus cereus* WU21001	17.33 ± 0.58	S	12.5/25	1/2^b^
*Staphylococcus aureus* ATCC25923	13.33 ± 0.29	S	12.5/50	0.500/1^b^

*^a^Ceftriaxone,

b Vancomycin, APEC=Avian pathogenic *Escherichia coli*, MIC=Minimum inhibitory concentration, MBC=Minimal bactericidal concentration, SD=Standard deviation

### Physicochemical characteristics and stability of microcapsules

#### Size and morphology of the microcapsule

**Figure-3:**
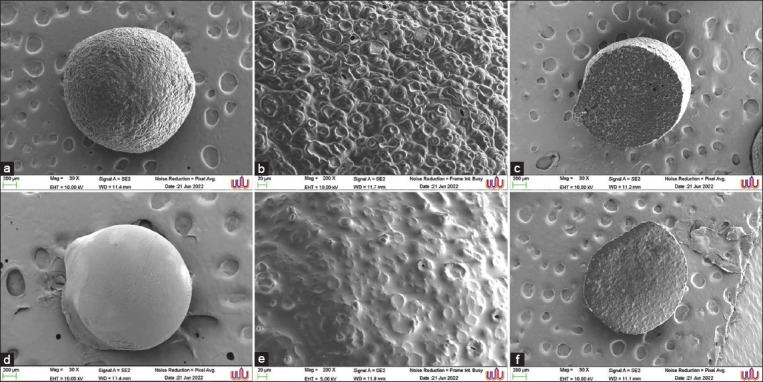
Scanning electron microscopy micrographs of (a) agar-alginate (AG-AL) microcapsule, (b) AG-AL microcapsule surface, (c) AG-AL microcapsule cross-section, (d) PAWU/AG-AL microcapsules, (e) PAWU/AG-AL microcapsule surface, and (f) PAWU/AG-AL microcapsule cross-section.

Based on the SEM images of the morphologies of the AG-AL and PAWU/AG-AL microcapsules, we observed significant porous networks on the AG-AL surface (Figures-3a and b). The diameters of the microcapsules ranged from 47 μm to 61 μm (Figure-3c). CaCl_2_ is known to create interconnected networks by ionic gelation with AG and AL. The addition of PAWU222001 smoothened the surface of AG-AL microcapsules (Figure-3d) to give PAWU/AG-AL (Figure-3e). The porous structure of PAWU/AG-AL was visible in the cross-section (Figure-3f), indicating that the porosity of AG-AL was maintained. The cross-sections showed a dense network, especially on the surface, which might belong to the AG-AL chains formed by hydrogen-bonding. The average diameters of PAWU/AG-AL were 500 μm, which was within the range of the feed ingredients (500 μm–2000 μm) [23]. The PAWU/AG-AL showed an EE percentage of 89.75 ± 0.1, representing successful microencapsulation.

#### Structural characterization of the microcapsules

**Figure-4:**
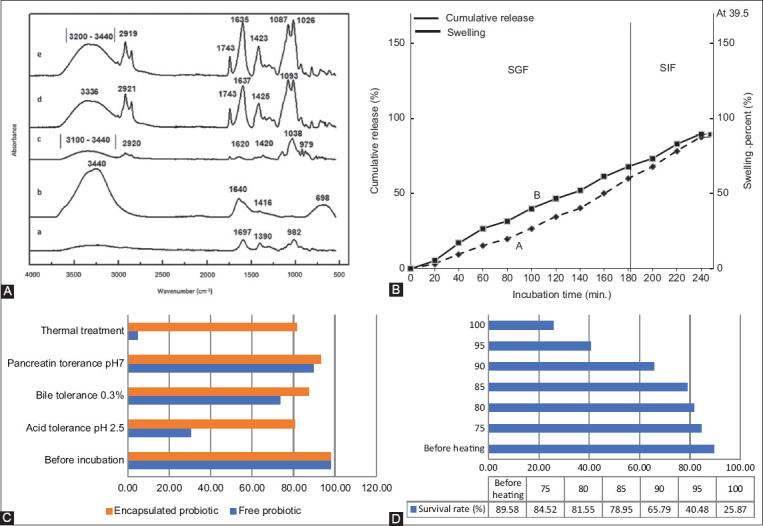
Fourier-transform infrared spectrometer spectra of (4A(a)) PAWU222001, (4A(b)) agar (AG), (4A(c)) alginate (AL), (4A(d)) AG-AL, (4A(e)). (B) PAWU/AG-AL (A), performances of PAWU/AG-AL in terms of swelling percent (%), and (B) cumulative release (%) in simulated gastric fluid and simulated intestinal fluid at 39.5 ± 0.5°C, (C) stability of free cells and PAWU/AG-AL under acid, bile, and trypsin tolerances including thermal treatment, (D) survival of PAWU222001 in AG-AL microcapsule after heating at different temperatures for 3 min.

Fourier-transform infrared spectrometer was used to analyze the functional groups and interactions between AG and AL in the microcapsules (Figure-4a). The typical O-H stretching peaks for AG and AL ranged between 3440 cm^-1^ and 3100 cm^-1^. After adding agar to the alginate matrix, this O-H peak displayed lower wavenumbers, indicating that hydrogen bonds might form between agar and alginate macromolecules (Figures-4A–D). A band was observed between 2921 cm^-1^ and 2919 cm^-1^ in all samples due to the ring of methine hydrogen atoms. Bands that appeared in all samples at approximately 1038 cm^-1^ and 939 cm^-1^ were mainly due to the coupling of the C-O stretching group, which is common to all polysaccharides (Figures-4A(b)-(c)). Furthermore, strong characteristic absorption peaks were observed around 1637 cm^-1^ in the AG/AL composite microcapsules, confirming the asymmetric and symmetric vibrations of the carboxyl group C=O.

Proteins with amino groups (NH_2_) and acidic carboxyl groups (COOH) are the main constituents of bacteria. The amino and carboxyl groups were confirmed at 900 cm^−1^–1300 cm^−1^ (Figure-4A(a). The absorption band appeared at 3440 cm^−1^–3200 cm^−1^ for PAWU/AG-AL (Figure-4A(e)), representing the stretching vibration of the intermolecular –OH group. The peak was slightly shifted, indicating that hydrogen bonds are involved in microcapsule formation. Furthermore, the peak intensity of the hydroxyl and carboxyl groups increased significantly, suggesting that PAWU222001 was successfully encapsulated in AG-AL.

Blending with natural polysaccharides requires high compatibility, and molecules can form hydrogen-bonding dipole-dipole forces or charge transfer complexes. Agar is a fibrous carbohydrate extracted from various marine algae from the class Rhodophyceae, also known as “red seaweed” [24]. It is composed of β-D-galactopyranosyl linked (1→4) to a 3, 6-anhydro-α-L-galactopyranosyl unit that is partially sulfated and produces perceptible gels at concentrations as low as 0.04% (Figure-1B(a)). The molecular chain of AG contains traces of sulfate groups. Alginate is a water-soluble linear anionic polysaccharide isolated from the cell walls of brown algae (*Laminaria digitate* and *Ascophyllum nodosum*) [25]. It is composed of monomeric units of 1→4-linked β-D-mannuronate (M) and α-L-guluronate (G) (Figure-1B(b)). In addition, AG and AL contain several hydroxyl groups, and AL also has a few carboxylic acid groups [19]. This facilitates the formation of intermolecular hydrogen bonds. Figure-1B(c) demonstrates the ideal gelation mechanism.

#### Swelling and cumulative release studies

Swelling and cumulative release studies were conducted to assess the release of PAWU222001 in the animal gut based on SIF and SGF. Figure-4B demonstrates the swelling as a function of incubation time. Simulated gastric fluid swelling values were 15.36% ± 1%, 34.56% ± 1.5%, and 60.24% ± 2% for the time intervals of 60, 120, and 180 min, respectively. The total weight of the PAWU/AG-AL microcapsules gradually increased to twice their initial weight. After 180 min in SIF, the average swelling of PAWU/AG-AL microcapsules was 87.56% ± 1.5%, indicating that AG-AL was responsive to the digestive fluid. During SGF treatment, acidic gastric pH (pH = 2–3) is expected to induce CaCl_2_ protonation, allowing water penetration, and swelling.

Treatment with SIF, which has a relatively neutral pH (6.5) but greater than the pKa of AG and AL (~3.6), caused deprotonation of AG and AL, initiating continuous swelling. Similarly, Corona-Hernandez *et al*. [23] reported that some carboxylate groups were ionized when the pH reached 6.5, increasing the swelling capacity.

The cumulative release of PAWU/AG-AL microcapsules shows a clear correlation between the swelling of PAWU/AG-AL microcapsules and the release of PAWU222001 (Figure-4B). At the beginning of incubation, the release was around 28% (60 min). The PAWU222001 on the AG-AL surface may have caused the initial burst release. At 180 min, the cumulative release of PAWU222001 in SGF was approximately 68%. If the pre-treatment to exclude burst release (28%) were performed, the release in SGF would be approximately 40%. At 240 min, the cumulative release in the SIF was approximately 90%.

The controlled release of a core substance depends on the type of polymer and its properties, such as degree of cross-linking, medium pH, mechanical forces, interaction with biological compounds or biological responses, and incubation time [26, 27]. Here, AG-AL works synergistically with the matrix material as follows. Initially, the ion exchange between calcium and chloride ions in SGF hindered the destruction of CaCl_2_ cross-linker. The erosion of microcapsule structure helps the SGF penetrate the AG-AL micropores. However, PAWU222001 was still maintained by the carboxylate groups of AG-AL chains and their networks in SGF, despite variations in pH and even endogenous enzymes in the gut. In the final step, the cross-linked network of AG-AL was gradually degraded as AG-AL deprotonated in SIF, causing the diffusion of PAWU222001. Similarly, Rather *et al*. [28] revealed that AL swelling occurred during ion exchange between calcium and phosphate ions in SIF. Probiotics were gradually released after the calcium ion-mediated cross-linked network was destroyed. According to the release mechanism described above, matrices and ionic cross-links of AG and AL should successfully release PAWU222001 in the lower part of the intestine.

#### Effect of acid, enzymes, and temperature on free and PAWU/AG-AL cell viability

When delivered *in vivo*, PAWU222001 must pass through several conditions, including acid, bile, and pancreatin. The roles of AG-AL in protecting PAWU222001 compared to free cells were investigated using bacterial cell count. Initially, PAWU222001 and PAWU/AG-AL demonstrated viability values of 99.5% ± 0.1% and 99.2% ± 0.5%, respectively (Figure-4C). When PAWU/AG-AL was incubated with acid, bile, pancreatin, and thermal treatment, the viability remained at approximately 80% in all cases. Furthermore, the viability of free cells significantly declined to 9%–94% when incubated with acid, bile, pancreatin, and thermal treatment (p < 0.05). The results indicated that protecting the matrices between AG and AL structures allowed significant viability. In other words, the viability of PAWU222001 reflects how matrices formed by AG and AL improve resistance to harsh environments and maintain viability. Zhang *et al*. [29] reported that encapsulation improves probiotic viability in gastrointestinal conditions. According to a study by Afzaal *et al*. [30], the microencapsulation of *Lacticaseibacillus casei* with calcium alginate and whey protein significantly improved probiotic viability in carrier foods and under GIT conditions.

#### Viability of PAWU/AG-AL under pelleting temperature

Typically, the manufacture of feed pellets requires temperatures between 80°C and 85°C [29]. Therefore, we investigated whether or not AG-AL encapsulated PAWU222001 improved cell viability at such temperatures. After treatment at 85°C, the viability of the naked PAWU222001 was as low as 20%–30%. Similarly, Gbassi *et al*. [19] revealed that free *Lactobacillus* spp. were 60% to 80% viable after incubation at 85°C. Figure-4D shows the viability of PAWU/AG-AL microcapsules after 3 min of heating at various temperatures. Initially, 89.65% of PAWU222001 were still viable. After 3 min of isothermal treatment at 75°C, 80°C, and 85°C, 80%–85% of PAWU222001 remained alive (p < 0.05). When the microcapsules were thermally treated at temperatures above 90°C, the viability of PAWU222001 decreased (65.37%), suggesting that microcapsules can benefit the feed pelleting process.

#### Effect of storage condition

Probiotics can be stored for a month before use in general applications. During this time, the viability might be lost due to the destruction of cell walls. The role of microcapsules is to maintain the probiotic survival rate and functional activities. We investigated the changes in the survival rate under storage conditions. The stability of probiotics is affected by various factors, including oxygen, light, moisture, temperature, and storage time [25]. These factors caused cell wall destruction, resulting in decreased performance and shelf life [30]. Table-2 shows that the survival of PAWU222001 gradually decreased over 30 days before dropping significantly to as low as 82%–100% at 90 days at 4°C or 28°C, respectively (p < 0.01). Meanwhile, the viability of PAWU/AG-AL showed almost no decline for 30 days at any storage time, while the viability slightly decreased (to 28%–33%) when stored for up to 90 days.

**Table-2:** Survival of PAWU222001 and PAWU/AG-AL expressed as a function of storage times at storage temperatures of 4°C and 28°C.

Sample	Storage time (day)	Survival amount at 4°C (%)	Survival amount at 28°C (%)
PAWU222001			
	0	91.17^a^ ± 0.21	89.24^a^ ± 0.09
	30	62.11^b^ ± 0.59	34.17^b^ ± 0.43
	60	27.86^c^ ± 0.87	3.63^c^ ± 0.76
	90	8.45^d^ ± 0.12	0^d^
PAWU/AG-AL			
	0	91.55^a^ ± 0.52	90.94^a^ ± 0.65
	30	87.12^a^ ± 0.86	85.73^a^ ± 0.43
	60	70.64 ^b^ ± 0.23	68.46^b^ ± 0.18
	90	63.19^c^ ± 0.57	57.51^c^ ± 0.76

*^a-d^Superscript for significant differences (p < 0.05) compared with before storage (day 0), AG-AL=Agar-alginate

## Conclusion

*Pediococcus acidilactici* WU222001, isolated from BX1, exhibited probiotic properties. The probiotic supernatant inhibited the growth of APEC, *E*. *coli*, *B*. *cereus*, and *S*. *aureus*, as revealed through agar well diffusion and MIC/MBC results. *Pediococcus acidilactici* was microencapsulated with sodium alginate and ranged from 47 mm to 61 mm in diameter. Importantly, microencapsulation enhanced bacterial viability in the GIT compared with free cells. Furthermore, 89.65% of the encapsulated cells were released into the SIF within 4 h. The viable count in microcapsules was 63.19% after 3 months of storage at 4°C. The results indicated that microencapsulation extended the viability of *P. acidilactici* under harsh conditions, indicating its potential application in the feed production.

## Authors’ Contributions

WM, SV, and WK: Conceived and designed the experiments. WM, SV, PS, JJ, TT, and SP: Performed the experiments, analyzed, and interpreted the data. WM, VN, MDLP, and SV: Wrote the manuscript. All authors have read, reviewed, and approved the final manuscript.
